# Bumble Bees (*Bombus* spp) along a Gradient of Increasing Urbanization

**DOI:** 10.1371/journal.pone.0005574

**Published:** 2009-05-15

**Authors:** Karin Ahrné, Jan Bengtsson, Thomas Elmqvist

**Affiliations:** 1 Department of Ecology, Swedish University of Agricultural Sciences, Uppsala, Sweden; 2 Department of Systems Ecology, Stockholm University, Stockholm, Sweden; University of Pretoria, South Africa

## Abstract

**Background:**

Bumble bees and other wild bees are important pollinators of wild flowers and several cultivated crop plants, and have declined in diversity and abundance during the last decades. The main cause of the decline is believed to be habitat destruction and fragmentation associated with urbanization and agricultural intensification. Urbanization is a process that involves dramatic and persistent changes of the landscape, increasing the amount of built-up areas while decreasing the amount of green areas. However, urban green areas can also provide suitable alternative habitats for wild bees.

**Methodology/Principal Findings:**

We studied bumble bees in allotment gardens, i.e. intensively managed flower rich green areas, along a gradient of urbanization from the inner city of Stockholm towards more rural (periurban) areas. Keeping habitat quality similar along the urbanization gradient allowed us to separate the effect of landscape change (e.g. proportion impervious surface) from variation in habitat quality. Bumble bee diversity (after rarefaction to 25 individuals) decreased with increasing urbanization, from around eight species on sites in more rural areas to between five and six species in urban allotment gardens. Bumble bee abundance and species composition were most affected by qualities related to the management of the allotment areas, such as local flower abundance. The variability in bumble bee visits between allotment gardens was higher in an urban than in a periurban context, particularly among small and long-tongued bumble bee species.

**Conclusions/Significance:**

Our results suggest that allotment gardens and other urban green areas can serve as important alternatives to natural habitats for many bumble bee species, but that the surrounding urban landscape influences how many species that will be present. The higher variability in abundance of certain species in the most urban areas may indicate a weaker reliability of the ecosystem service pollination in areas strongly influenced by human activity.

## Introduction

Bumble bees and other wild bees play an integral ecological role as pollinators of a large number of wild flowers and cultivated crops [1]–[4]. There has been a documented decline in diversity and abundance of wild bees in Europe and the United States during the last decades. The main causes of this decline are considered to be habitat destruction and fragmentation as consequences of human activity [5]–[7]. Still our understanding of the response of wild bees to habitat fragmentation is rather limited [8]–[10]. Human activities, such as increasing urbanization and agricultural intensification, imply extensive modifications of the landscape and the environment and lead to destruction and fragmentation of natural habitats. Urbanization is among the most important human activities that cause drastic and persistent alteration of habitats, and it is likely to increase in the future [11]. Buildings, roads, and industrial areas, together often termed impervious surfaces, increase with a corresponding decrease of green areas.

In this study we focus on the response of bumble bees to increasing urbanization. Understanding the effects of urbanization on bees is important for several reasons. Firstly, the loss and isolation of existing habitats due to urbanization may affect bees negatively. In a study of bees and wasps in Belo Horizonte (Brazil), the abundance of eusocial stingless bees was directly negatively affected by the loss of vegetation cover and the increase of buildings associated with urbanization [12]. In addition, the loss of vegetation cover had a negative effect on the abundance and species richness of advanced eusocial wasps. Secondly, urban areas also include flower rich green areas that may still harbour a high diversity and abundance of wild bees. Some examples of such areas that merit attention are allotment, private and botanical gardens, city parks, road verges and other types of ruderal areas. For instance, although overall bumble bee species richness in the San Francisco area has declined, urban parks were as diverse and had higher abundances of bumble bees than nearby wilder parks [13]. Another study documented 262 bee species in disturbed and ruderal areas in the city of Berlin [14]. Thirdly, urbanization may affect different bumble bee species differently. Several bumble bee species in Europe and North America have declined and become locally extinct, while other species still are widespread and common [15]. The causes of this difference in response are not clear, but it has been attributed to species specific traits such as tongue length [16], diet [17], species' geographical ranges [18], emergence time [19] and foraging distance [20]. Differences in foraging distances have been explained by differences in body [21], [22] or colony size [21].

Our study aimed at examining the relative importance of landscape structure and habitat quality for local diversity and abundance of bumble bees, along an urban-periurban gradient in Stockholm, Sweden's largest urban area with about 1.4 million inhabitants. The gradient was *a priori* defined by the amount of impervious surface within the surrounding landscape. Bumble bee diversity and abundance were studied in 16 allotment gardens, which are areas reserved for horticulture where plots are let to individuals for growing, e.g., vegetables and flowers for non-commercial use. Allotment gardens are found in central Stockholm as well as in periurban areas at the outskirts of the city. These gardens are generally intensively managed flower rich green areas, thus potentially good habitats for bumble bees. Among different urban habitats in Vancouver (Canada), botanical and community gardens (with management similar to allotment gardens) had the highest abundances of bees [23]. Likewise, allotment gardens in Stockholm had more species and much higher abundances of bumble bees than two other common types of green areas (parks and cemeteries) [24]. In contrast to earlier studies of bees in urban areas [13], [23], [25], [26] all our inventories were done in the same type of flower rich habitats along the gradient of increasing urbanization, thus avoiding confounding effects of different habitats and urbanization.

Low-diversity communities are expected to vary more in their functioning than high-diversity communities [27]. Therefore, if bumble bee diversity is negatively affected by urbanization, the variation in the function, i.e., pollination (here flower visits), may be higher in an urban than in a more rural setting. The variability in flower visits has implications for the reliability of the ecosystem service pollination [28].

We address the following hypotheses: bumble bee diversity and abundance are (1) negatively related to increasing urbanization, measured as increasing proportion of impervious surfaces in the landscape surrounding the studied sites, and (2) positively related to increasing flower richness and abundance in the allotment gardens; (3) the variation in flower visits by bumble bees is higher between allotment gardens surrounded by a high proportion of impervious surface compared to those in a more rural setting.

## Results

### Bumble bees

Bumble bees were surveyed in study plots (triangles of 3×3 meters) distributed within the allotment garden to contain flowers in bloom (see Material and methods for details). In total 1937 bumble bee individuals of 13 species were observed (see Table 1). Two of the species were cuckoo bumble bees; *B. bohemicus and B. rupestris*, which are nest parasites of other bumble bees. The number of species observed per allotment garden ranged between 5 and 11. Seven species, *B. hypnorum*, *B. lucorum*, *B. terrestris*, *B. lapidarius*, *B. pascuorum*, *B. hortorum* and *B. ruderarius*, were observed at most sites (at least 14 of 16). Among the species that were scarce, occurring in less than half of the sites, were *B. subterraneus*, *B. soroëensis* and *B. sylvarum* and the cuckoo bumble bees, *B. bohemicus*, and *B. rupestris*.

**Table 1:** Bumble bee species recorded in the study.

Bumble bee species	Mean no. ind. found, n = 16 (SE)	Tongue length	Size
*B. hortorum* (L.)	0.17 (0.06)	Lt	L
*B. hypnorum* (L.)	0.24 (0.04)	St	L
*B. lapidarius* (L.)	0.38 (0.06)	St	L
*B. lucorum* (L.)	0.30 (0.06)	St	L
*B. pascuorum* (Scop.)	0.26 (0.06)	Lt	S
*B. pratorum* (L.)	0.10 (0.04)	St	S
*B. ruderarius* (Müller)	0.62 (0.13	Lt	S
*B. soroëensis* (Fabr.)	0.02 (0.01)	St	S
*B. subterraneus* (L.)	0.01 (0.00)	Lt	L
*B. sylvarum* (L.)	0.03 (0.01)	Lt	S
*B. terrestris* (L.)	0.67 (0.10)	St	L
*B. bohemicus* (Seidl)	0.02 (0.01)	-	-
*B. rupestris* (Fabr.)	0.02 (0.01)	-	-

The bumble bee species found in the 16 allotment gardens, the mean number of individuals of each species per site, plot and 5 min (SE within parenthesis) and their respective size and tongue length categories, Lt = Long tongue, St = Short tongue, L = Large, S = Small. The cuckoo bumble bees *B. bohemicus* and *B. rupestris* were not included in the tongue length and size categories. Bumble bees were divided into two groups based on tongue length according to [16], [for B. hypnorum 54]. They were also divided into two size groups based on information on body size [44].

Bumble bees were observed on a total of 168 plant species. The most visited plant families were Lamiaceae, Asteraceae, Fabaceae, Boraginaceae and Malvaceae. Fifty percent of the total number of visits was to fourteen species. These were, in decreasing order, *Origanum vulgare*, *Solidago gigantea*, *Rubus ideaus*, *Astrantia major*, *Centaurea montana*, *Malva* spp., *Lavendula augustifolia*, *Coreopsis verticillata*, *Sedum spurium*, *Impatiens glandulifera*, *Aconitum*×stoerkianum, *Lythrum salicaria*, *Symphytum*×uplandica and *Centaurea cyanus*. *O. vulgare* was a common plant species at all allotment areas and was visited by most bumble bee species.

### Diversity and abundance

**Figure 1:**
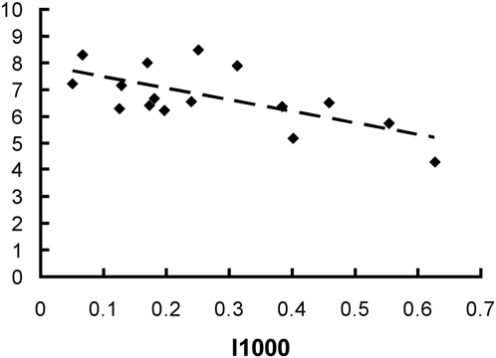
Effect of impervious surface on bumble bee diversity. The relationship between bumble bee diversity (number of species after rarefaction to 25 individuals) and proportion impervious surface within 1000 m.

Species diversity of bumble bees was measured after rarefaction to 25 individuals, which accounts for variation in the number of observed bumble bees between sites. Diversity was negatively related to the amount of impervious surface (I) within a radius of 300, 500 or 1000 m, and consequently also related to several other variables strongly correlated with I, such as proportion of forest and length of boundaries towards forest (both positively related to diversity; Table S1). The proportion of impervious surface was the variable explaining most of the variation in species diversity within all three radii. Other variables included in the analysis did not have significant effects on diversity: the proportion of urban green areas (G), the size of allotment garden (Size), and mean flower abundance within study plots (Flower cover) (see Table 2 for all variables measured and Table S1 for the full correlation matrix). The strongest correlation between bumble bee diversity and the proportion of impervious surface was found for the 1000 m radius (Figure 1), but the same pattern was found for the other two radii as well (Table 3). The results were essentially the same when examining the unrarefied species richness. For 300 m and 1000 m radii the proportion of impervious surface was the variable explaining most of the variation, while for 500 m radius none of the variables examined was significant (Table 3).

**Table 2:** Landscape and local variables measured.

Scale	Variable type	Abbreviations and measures
Landscape(300 m, 500 m and 1000 m radii)	Land cover	**I** = Proportion of impervious surfaces, e.g. buildings, roads, industrial areas.
		**G** = Proportion of green areas, e.g. parks, gardens, pastures, golf courses.
		**F** = Proportion of forest.
		**A** = Proportion of arable land.
	Boundaries towards different land cover types	**GB** = Length of green area boundary/unit area.
		**FB** = Length of forest boundary/unit area.
		**WB** = Length of water boundary/unit area.
		**AB** = Length of cultivated land boundary/unit area.
		**PB** = Length of pasture boundary/unit area.
Local (allotment garden)	Flower richness	**Cov1** = Percent cover of flowering plants in 5 m×5 m plots in July.
		**Cov2** = Percent cover of flowering plants in 10 m×10 m plots in August.
		**Plant sp1** = Number of flowering plant species 5 m×5 m in July.
		**Plant sp2** = Number of flowering plant species 10 m×10 m in August.
		**Flower cover** = Percent cover of flowering plants in small study plots (triangles).
		**Plant sp/small plot** = Number of plant species/study plot (triangles).
	Type of site	**House** = Allotment gardens with plots with houses.
		**Mixed** = Allotment gardens with plots with and without houses.
		**Garden** = Allotment gardens with plots without houses.
	Age	**Age** = Years since establishment of the allotment.
	Size	**Size** = Area covered by the allotment (m^2^).

**Table 3:** Step-wise regressions of species diversity and total abundance.

Dependent variable	Radius (m)	Independent variable	F model	p model	r^2^ model
Species diversity	300	I300	7.26	0.017	29.4
	500	I500	4.72	0.048	19.9
	1000	I1000	9.68	0.008	36.7
Un-rarefied raw data	300	I300	6.48	0.023	26.8
	500	I500			
	1000	I1000	7.20	0.018	29.2
Bumble bee abundance	300, 500 and 1000	Flower cover	>5.02	<0.042	>21.1

Results of step-wise regressions with species diversity, bumble bee abundance and unrarefied raw-data as dependent variables and Flower cover, Size, I (300, 500 or 1000 m) and G (300, 500 or 1000 m) as independent variables.

Bumble bee total abundance was positively related to flower resources. The variable explaining most of the variation in bumble bee abundance was the proportion of flowering plants within study plots (Flower cover) (Table 3).

### Effect of type of allotment garden on bumble bee diversity and abundance

Allotment gardens without houses had approximately one more species than the other two types of sites (Garden vs. Mixed or House), but there was no interacting effect of allotment garden type and urbanization on neither bumble bee diversity nor abundance. Analyses of Covariance (ANCOVAs) showed no interactions between type of site and impervious surface, at any of the three spatial scales (Diversity: I300: F = 0.06, p = 0.945, df = 2, I500: F = 0.31, p = 0.740, df = 2, I1000: F = 0.49, p = 0.62, df = 2; Abundance: I300: F = 1.17, p = 0.349, df = 2, I500: F = 0.52, p = 0.608, df = 2, I1000: F = 0.88, p = 0.443, df = 2). When we accounted for the effect of impervious surface there was a significant (p<0.05) effect of type of site on bumble bee diversity at all scales (Table 4), but not on bumble bee abundance. Plant species richness and abundance did not differ significantly between the three types of site.

**Table 4:** Analyses of covariance.

	Independent variables	F	p	r^2^ model
Model	Type of site	6.93	0.010	61.8
Covariate	I300	9.80	0.009	
Model	Type of site	6.26	0.014	54.2
Covariate	I500	6.19	0.028	
Model	Type of site	5.73	0.018	62.2
Covariate	I1000	10.02	0.008	

Result of ANCOVAs with species diversity (rarefied data to 25 inds.) as dependent variable, type of site as explanatory factor and I (300, 500 or 1000 m) as covariates.

### Species composition

**Figure 2:**
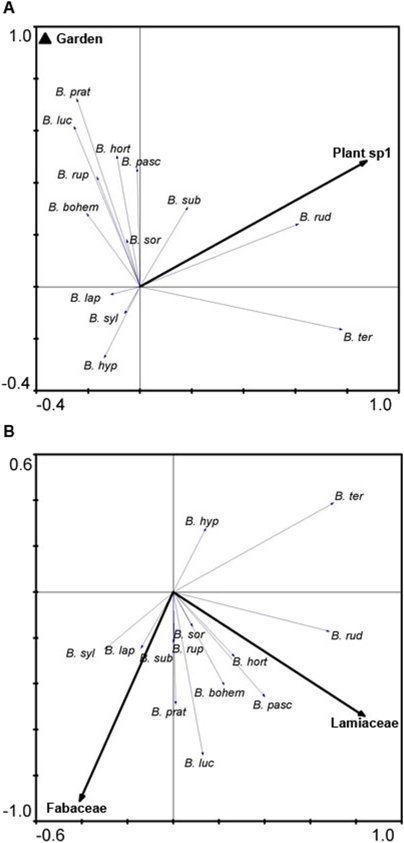
Effect of habitat variables and plant families on species composition. (A) Results from RDA with habitat variables as explanatory variables. Together the first two axes explain 40.9% of the variation in bumble bee species composition (Eigenvalues: axis 1 = 0.291, axis 2 = 0.118). The two most important habitat variables were: The number of flower plant species (Plant sp. 1; p = 0.003, F-value = 4.677) and the Garden type of site (p = 0.007, F-value = 3.479). Species abbreviations: *B. bohem = B. bohemicus*, *B. hort = B. hortorum*, *B. hyp = B. hypnorum*, *B. lap = B. lapidarius*, *B. luc = B. lucorum*, *B. pasc = B. pascuorum*, *B. prat = B. pratorum*, *B. rud = B. ruderarius*, *B. rup = B. rupestris*, *B. sor* = *B. soroëensis*, *B. sub = B. subterraneus*, *B. syl = B. sylvarum*, *B. ter = B. terrestris*. (B) RDA with plant families as explanatory variables. Together the first two axes explain 43.6% of the variation in bumble bee species data (Eigenvalues: axis 1 = 0.309, axis 2 = 0.127). The two most important plant families were: Lamiaceae (p = 0.001, F = 4.798) and Fabaceae (p = 0.003, F = 4.166).

Bumble bee species composition was examined using Redundancy Analysis (RDA) with habitat variables as explanatory variables. The first two axes in the RDA together explained 40.9% of the variation in bumble bee species composition. Two habitat variables, the number of flowering plant species within 5 m^2^ (Plant sp. 1) and the Garden type of site were significant (Plant sp. 1: p = 0.003, F = 4.68, Garden: p = 0.007, F = 3.48). The bumble bee species *B. ruderarius* and to some extent *B. subterraneus* were positively related to the number of flowering plant species, whereas *B. hortorum*, *B. lucorum*, *B. pascuorum*, *B. pratorum* and *B. soroëensis* and the cuckoo bumble bees *B. bohemicus* and *B. rupestris* were all more abundant on garden sites (Figure 2A). None of the landscape variables significantly explained variation in bumble bee species composition at any of the three spatial scales.

In a RDA with plant families as explanatory variables the first and second axes accounted for 30.9% and 12.7%, respectively, of the variation in bumble bee species data. The first axis was best related to the abundance of plant species belonging to the family Lamiaceae and the second axis was best related to Fabaceae (Figure 2B).

### Variability in bumble bee observations

**Figure 3:**
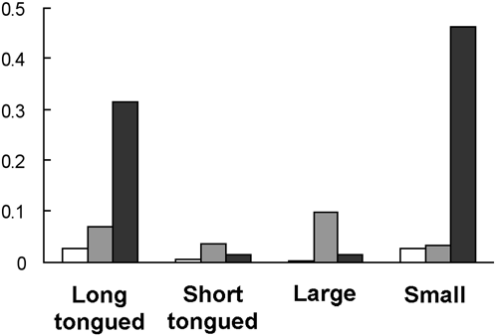
Variation in bumble bee abundance. Variances between sites in log mean visits/plot/5 min. Bartlett's F-test showed that significant differences in variance of log mean values were found for *Long tongued species* (Periurban vs. Urban; p = 0.041) and for *Small species* (Periurban vs. Urban; p = 0.02. Intermediate vs. Urban; p = 0.015). White bars = periurban sites, grey bars = intermediate sites, black bars = urban sites.

For the analysis of spatial variability in abundance, the bumble bee species were divided into groups based on their average body size and tongue length (Table 1) and the allotment gardens were divided into three groups (periurban, intermediate and urban) based on the proportion of impervious surface within the surroundings (see Material and methods for details). Differences in variability between sites within groups were analysed with Bartlett's F-test. The mean number of bumble bees observed did not differ significantly between periurban, intermediate and urban sites for any of the four species categories long-tongued, short-tongued, large or small species. However, the spatial variability in the number of bumble bee observations per plot and unit time differed significantly between the three groups for small species (p<0.01) and near significantly for long-tongued species (p = 0.073). In pair-wise comparisons the variability in small species observations was significantly higher in urban sites than in the other two types of site, and the variability in long-tongued species observations was significantly higher in urban than in periurban sites (Figure 3). For short-tongued species no significant difference in variability between the three groups was found (p = 0.256). For large species Bartlett's F-test was not applicable as the data were not normally distributed. When analysing all bumble bees together, neither the mean number of individuals observed nor the variability in the number of bumble bee observations per plot differed significantly. However, there was a tendency for variability to be higher in intermediate and urban sites compared to periurban sites (Periurban vs. Intermediate, p = 0.074, Periurban vs. Urban, p = 0.11; variance for Periurban = 0.0059, Intermediate = 0.044 and Urban = 0.036).

## Discussion

We found that bumble bee diversity was negatively affected by urbanization as measured by increasing proportion of impervious surface within the surrounding landscape. Bumble bee abundance and bumble bee species composition, on the other hand, were most affected by site-specific characteristics such as flower abundance or plant species richness. The negative effect on diversity of increasing proportion of impervious surface also represents the effect of decreasing proportions of forest and arable land, and decreasing length of forest, field or pasture boundaries within the landscape. That is, proportions of areas with suitable nesting sites and alternative foraging sites decrease with increasing urbanization. Allotment gardens can provide forage for a large number of bumble bee species, but the surrounding urban landscape determines which and how many species that will occur.

Urban parks in San Francisco were found to have as diverse bumble bee assemblages as nearby larger wild parks [13]. However, an overall decline in bumble bee diversity in the area was also found, and only four out of nine bumble bee species historically found in the city were recorded. This study, as well as several other studies on wild bees [23], [25], [26], have compared urban habitats with different wilder habitats. Here we show that diversity of bumble bees is lower in an urban than in a more rural setting also when studying the same type of habitat, namely allotment gardens. Thus, transformations of the landscape associated with urbanization *per se*, here represented by an increasing proportion of impervious surface, affect bumble bee diversity negatively. Our results suggest that bumble bee abundance, on the other hand, is more affected by local factors, such as flower abundance, than by the surrounding landscape. Similarly, other studies have found that urban parks had higher abundance of bumble bees than nearby wilder parks [13], and that bee abundance was higher in flower rich botanical and community gardens than in wild areas [23].

Previous studies of the effect of landscape context on flower visitors in the agricultural landscape in Germany [9] and in the Netherlands [29] suggested that bumble bee diversity and abundance may not be significantly affected by the surrounding landscape. In our study total bumble bee abundance was not affected by the surrounding landscape, but bumble bee diversity, also when controlling for abundance differences by rarefaction, was significantly influenced by the landscape context at all three spatial scales measured. However, it was not possible to separate the importance of the landscape at the three different scales in our study, because the landscape variables were highly correlated between the three radii. One explanation to the differing results could be that bumble bees react differently to the landscape context in different types of landscape, i.e., agricultural versus urban. Another and in our view more likely explanation may be that the landscapes surveyed in Germany and the Netherlands are already so intensively influenced by human activities that the most sensitive bumble bee species are no longer present. In our study 13 bumble bee species were observed while in the Netherlands only four very common species were observed, and in Germany eight species. The differences between our results and the results of the two other studies suggest that one should be cautious when drawing conclusions about bumble bees in general by surveying the most common species only see also e.g. [30].

Small and long-tongued species were on average as abundant in urban as in periurban and intermediate sites, but the variability in abundance among sites was significantly higher in urban than in periurban or intermediate sites. This finding may have implications for the reliability for pollination as an ecosystem service. Many plant species with long corollas are mainly visited and successfully pollinated by long-tongued bumble bee species [1], [31]. Long-tongued bees showed significantly higher spatial variability among the urban sites than among the periurban. The higher variability was mainly caused by *B. ruderarius*, which was very common in two relatively closely situated urban sites but was not found at all in another urban site. This species was also observed to be less common in 2005, when it was subject to another study [24]. Thus, it may be a species that varies in abundance between years as well as between sites. The other small species all showed a tendency to be less common in allotment gardens in the most urbanized areas. *B. sylvarum* was not found at all in any of the seven sites with a proportion of impervious surface larger than 17% within 300 m radius and only one single individual of *B. pratorum* was found in one of the six sites with a proportion of impervious surface larger than 18% within 300 m radius. *B. soroëensis* was also absent from the four most urban sites. Thus, urbanization seems to affect several small species negatively. *B. ruderarius*, *B. sylvarum* and *B. soroëensis* are species that are declining elsewhere in Europe [15], [20], whereas *B. pratorum* is considered among the ubiquitous species not currently in danger [20]. No large species was negatively influenced by impervious surface and they were usually found in at least 14 of 16 sites. The exception was *B. subterraneus*, which was only found sporadically in five allotment gardens.

Bumble bees need two main resources: (1) nectar and pollen rewarding flowering plants and (2) suitable nesting sites within their foraging range. Foraging distances among bumble bees have been debated and it is not clear whether bumble bees prefer to forage close to their nests reviewed in [15], [32] or at a distance, both have been suggested [33], [34]. Nonetheless, different species certainly have different foraging ranges e.g. [35]–[37], which suggests that the landscape surrounding foraging and nesting sites will affect different species differently. Movement of bumble bees in urban areas may also be hindered by human structures such as roads and railroads [10]. Previous studies of foraging distances among bees have found it to be influenced by body size [22]. For example, foraging distance and forage trip duration for solitary bees were correlated with body length [38]. Also among bumble bees large species have generally been found to be able to fly further than small species [30], [35], [39] but see [37]. However, the flight distances of most bumble bee species remain unstudied.

We surveyed bumble bees while foraging and even though nests of at least three species, *B. terrestris*, *B. hypnorum* and *B. lapidarius*, were found within the allotment gardens no actual estimates of the number of nests within the sites could be done due to the well-known difficulties in finding bumble bee nests. However, clearly bumble bees with short foraging distances will have to nest within or in the close vicinity to the allotment gardens whereas bumble bees with large foraging ranges can nest further away and still utilize the flower resources within the allotment garden.

Species composition was most affected by local factors such as flower richness and type of allotment garden. Most species increased with increasing flower richness and were more abundant in allotment gardens with cultivated plots only. The most influential plant families were Lamiaceae and Fabaceae. This was probably due to the high number of bumble bees visting *Origanum vulgare* and other aromatic plants such as *Nepeta cataria*, *Lavendula angustifolia*, and *Salvia* spp., which are all commonly grown in the allotment gardens. Other important plant species of the family Lamiaceae found in the allotment gardens were *Stachys byzantina* and *Lamium album*. Both *Lavendula angustifolia* and *Lamium album* have been described as visited by a wide range of bumble bee species [40]. The latter was also found to be visited by queens in the spring and favored by workers of long-tongued bumble bees later in the season. Fabaceae, and *Trifolium pratense* in particular, is an important source of pollen and nectar especially for many long-tongued bumble bee species [16], [41]. Other species belonging to the family Fabaceae commonly found in the allotment gardens were *Lupinus* spp., *Vicia* spp., and *Trifolium repens*.

Although urbanization negatively affected species diversity of bumble bees in the studied allotment gardens, the total number of species observed in our study, 13 out of the 22 species present in the region [42], was rather high. Even the five most urban sites had 11 species together. The species that were not found by us can either be classified as threatened or rare or are cuckoo bumble bees. The latter are dependent on the presence of their host species and in general have smaller colonies than other bumble bees. *B. subterraneus*, which was found in five allotment gardens, is declining in many other countries in Europe [16], [20] and has only recently been removed from the Swedish red-list of endangered species.

Taken together, our results suggest that allotment gardens can provide forage for many bumble bee species, but that the surrounding urban landscape influences how many species that will be present. Together with other studies of bees in urban areas [14], [23], [26] this indicates that urban green areas can serve as important alternatives to natural habitats for bees and bumble bees. Nevertheless, it is clear that both the local management of the urban green areas, see also [24], [25], and the future planning and sprawl of the city will influence the diversity and abundance of bumble bees. The higher variability in abundance of certain species in the most urban sites suggests that ecosystem services such as pollination may be less reliable in urban and other areas strongly influenced by human activities, see also [28]. In the light of declining diversity and abundance of important pollinators such as bumble bees both elsewhere in Europe and in the United States our results are both encouraging and demanding. To support a relatively high number of bumble bee species in the future, urban planners must become aware of the importance of areas with high diversity, such as allotment gardens, and also actively plan the larger urban landscape to maintain pollination services.

## Materials and Methods

### Study sites

The study was located in Stockholm City and in surrounding northern municipalities, together with approximately 1.4 million inhabitants [43]. This is one of the most densely populated areas in Sweden. Sixteen allotment gardens situated along an urban to periurban gradient, from the inner city of Stockholm approximately 30 km towards the north and more rural areas, were chosen as study sites (59°18′–59°38′N, 17°43′–18°05′E). The allotment gardens differed in appearance, some with plots only used for cultivation, while others also had small houses and lawns. Therefore the different types of allotment gardens were divided into three groups for some of the analyses: those with cultivated plots only (Garden), those containing some plots with small houses (Mixed) and those with small houses on almost all plots (House). The allotment gardens also differed in size, 3450–70 000 m^2^, and were established at different times from 1905 to 1985. Despite these differences they could all be characterised as intensively managed flower rich green areas. The allotment gardens were rich in flowers during the whole study period and were among the most flower rich areas within 1000 m radius.

### Bumble bee survey

Daylight (9.00 am to 19.00 pm) surveys of bumble bees were done in July and August 2003 in good weather (temperature>15°C, sunny or scattered clouds). Each allotment garden was visited four times in varying order so that all sites were surveyed both in the morning and in the afternoon. At each site, point observations of bumble bees were conducted at 5 to 12 study plots, consisting of a triangle with sides three meters. The number of plots depended on the size of the site and was related to the logarithm of the area. The plots were evenly distributed within the allotment gardens and placed to contain plant species in flower. During bumble bee surveys the observer was standing in one of the corners of the triangle. All bumble bees entering the study plot during a five minute survey period were identified to species according to [44] and the plant species visited was recorded. When species determination was not possible by sight, bumble bees were caught with a net and either determined to species on site or brought to the laboratory for later determination. The five minutes were measured with a stopwatch that was temporarily stopped while catching a bumble bee.

### Quantifying site and landscape structure

To describe the flower richness of the sites, the coverage of flowering plants was estimated in the study plots (triangles with sides 3 m). Within each plot, percentage cover of flowering plants as well as the plant species and cover of each species were noted. In addition, to get a more accurate description of the local flower richness and composition, percentage cover and species of flowering plants were noted in larger quadrats of 5 m^2^ (in July) and in 10 m^2^ (in August) placed at each study plot. Flowers were identified to species following [45], [46]. The overall mean cover of flowering plants within allotment gardens was 29% (±S.E.1.5) within 3 m×3 m triangles, 36% (±S.E.2.1) within 5 m×5 m quadrats and 24% (±S.E.1.7) within 10 m×10 m quadrats. The mean number of flowering plant species/triangle or quadrat was 3.1 (±S.E.0.15) within 3 m×3 m triangles, 8.1 (±S.E.0.64) within 5 m×5 m quadrats and 11.2 (±S.E.0.99) within 10 m×10 m quadrats.

To characterize the landscape surrounding each study site we used the Swedish CORINE Land Cover Data (GSD-Marktäckedata), obtained from the Swedish Land Surveying Authority (Lantmäteriet). The Land Cover Data is a database with information on land use and vegetation with mapping units of 25 m×25 m. It is based on the European mapping CORINE Land Cover, a database that is homogeneous all over Europe (www.lantmateriet.se). The landscape characteristics were analysed at three radii, 300, 500 and 1000 m centred at the midpoint of each allotment garden. These radii were chosen because estimated flight distances of bumble bees are between a few hundred meters for some species to some kilometres for others [22], [33], [35], [47], [48]. Three hundred meters was used as the smallest radius to describe the surrounding landscape as smaller radii would, in most cases, have included only the allotment gardens. The proportion of different land-cover types, i.e., impervious surfaces (e.g. roads, buildings, and industrial areas), arable land, forest and other green areas (e.g. pastures, gardens, city parks) for these three radii were quantified using the Geographic Information Systems ArcGIS 9.0. There was a clear gradient in the proportion of impervious surface surrounding the allotment gardens. It ranged between 0–53, 3–69 and 5–62 percent within the radii 300, 500 and 1000 m respectively. Within the three radii the total length of boundaries between different types of land-cover was measured, as especially forest boundaries and field margins are known to be important both for nest searching and foraging bumble bees [49]–[51]. To account for possible differences in time since establishment and area of the allotment garden, these variables were also included in the analyses (see Table 2).

### Statistical analysis

Because the number of bumble bee individuals observed differed among sites, partly because of different number of study plots at different sites, it was useful to standardise the number of species to a certain sample size. Therefore, an individual-based rarefaction on the bumble bee data was done in EcoSim 7.71 [52]. Individual-based rarefaction uses probability theory to compute the expected number of species at each site if the same number of individuals would have been observed at each site. In our case the lowest number of individuals observed at a site was 25. Thus, we used the expected number of species when drawing 25 individuals from each site in our analyses. As the rarefied number of species depends on the distribution of individuals among different species [53] it can be interpreted as a measure of diversity and from now on when speaking of bumble bee diversity we mean the rarefied number of species. However, because 25 individuals might be a too small sample to detect differences in diversity [53] we also analysed the un-rarefied raw data, to ensure that the rarefaction did not have such an effect on the results reported.

A stepwise regression was done to find the variables explaining most of the variation in species diversity and abundance. To decide which variables to use in the stepwise regression we first calculated the full correlation matrix (Table S1). When variables were strongly correlated with each other and often probably described the same gradient, only one of them was chosen for the final analysis (see Table S1 for further information). As the measurements at the three different radii were always strongly correlated (p<0.05), the radii were analysed separately. For example, the proportion of impervious surface within 300 m was correlated with the proportion of impervious surface within 1000 m (r = 0.93). All proportions were Arcsine Square root-transformed and garden size was log-transformed before the analyses.

After the stepwise regression analyses, we examined the effect of type of site (Garden, Mixed and House) and impervious surface on bumble bee diversity and abundance by ANCOVAs. One-way ANOVAs were performed to test if number of plant species or coverage of flowering plants differed between different types of site. Stepwise regressions, ANCOVAs and ANOVAs were done using MINITAB 14.

Differences in bumble bee community composition explained by the environmental data available were analysed with constrained ordination in CANOCO 4.5. The gradient length of the species data was first tested using Detrended Correspondence Analysis (DCA). As the gradient length was short (1.77 SD units) indicating a linear response, Redundancy Analysis (RDA) was used. The three radii were analysed separately. The effect of habitat and landscape variables was first analysed separately to identify the most important habitat variables and the most important landscape variables within each radius using Forward Selection. The level of significance was estimated with 999 Monte Carlo permutations.

To investigate in more detail how and which components of flower richness and abundance affected the variation in bumble bee species composition, we did another RDA using the percent cover of different plant families in small plots as explanatory variables. Plant families that occurred in at least 14 of the 16 allotment gardens were included in the analysis; 11 plant families in all.

To assess the variability in bumble bee flower visits between allotment gardens along the gradient the 16 sites were divided into three groups; periurban (five sites surrounded by less than 10% impervious surface within 300 m), intermediate (six sites surrounded by 11–20% impervious surface within 300 m) and urban sites (five sites surrounded by 25–53% impervious surface within 300 m). The mean number of bumble bees observed per plot per 5 minutes was first calculated for each allotment garden. Then the variability in bumble bee observations between sites within the three groups was analysed. Bumble bees were divided into two functional groups: long-tongued species and short-tongued species according to [16], [54] (see Table 1). They were also divided into two size groups according to their average body sizes, i.e., small species or large species according to [44], (see Table 1). Even if there is large variation within species both in tongue length and in body size it is possible to divide the species into groups from species specific average measures of these characteristics. It has been suggested that long-tongued bumblebees have more specialized diets [16] and that these species have declined more than short-tongued bumble bees [1], which makes this distinction interesting. Further, it appears that body size affects bee foraging ranges [22] and probably the scale at which they perceive and react to the landscape [21]. Spatial variability in bumble bee observations was analysed in each of the four groups. All species, except cuckoo bumble bees, were included in two analyses - one examining body size and one examining tongue length. Homogeneity of variances of the log mean number of observations/plot and 5 min were tested with Bartlett's F-test according to [55], [56].

## Supporting Information

Table S1 The full correlation matrix of all variables.
(0.19 MB DOC)
