# Supplementary material for: Bumble Bees (Bombus spp) along a Gradient of Increasing Urbanization
Source: PLoS One. 2009 May 15;4(5):e5574. doi: 10.1371/journal.pone.0005574 (PMC2679196; doi:10.1371/journal.pone.0005574)
Supplement: Table S1 — The full correlation matrix of all variables. (0.19 MB DOC) [file pone.0005574.s001.doc]

**Supporting information**

**Correlations between local and landscape variables**

Local and landscape variables entering the model were correlated with other independent variables not entering the model as follows [Figure S1]: The proportion of impervious surface within all three radii was negatively correlated with proportion of forest and arable land, length of forest, field and pasture boundaries, and positively correlated with age of the allotment gardens. The proportion of green areas within all three radii was negatively correlated with proportion of forest, length of forest and field boundary, and positively correlated with length of boundary towards green areas. Because of these correlations, we did not use measures of boundaries in the analyses, as they were accounted for by the included variables. The percent cover of flowering plants in bumble bee study plots (Flower cover) was correlated with a number of other variables describing flower richness in the allotment gardens.

**Table S1** The Full Correlation Matrix of all Variables

|  | Flower cov | Cov 1 | Cov 2 | Plant / plot | Plants 1 | Plants 2 | I300 | G300 | F300 | A300 | I500 | G500 | F500 | A500 | I1000 | G1000 | F1000 | A1000 |
| --- | --- | --- | --- | --- | --- | --- | --- | --- | --- | --- | --- | --- | --- | --- | --- | --- | --- | --- |
| Cov 1 | 0.31 |  |  |  |  |  |  |  |  |  |  |  |  |  |  |  |  |  |
| Cov 2 | 0.17 | 0.04 |  |  |  |  |  |  |  |  |  |  |  |  |  |  |  |  |
| Plant / plot | **0.79** | 0.46 | 0.13 |  |  |  |  |  |  |  |  |  |  |  |  |  |  |  |
| Plants 1 | **0.51** | **0.64** | 0.14 | **0.72** |  |  |  |  |  |  |  |  |  |  |  |  |  |  |
| Plants 2 | **0.70** | 0.29 | 0.36 | **0.62** | 0.49 |  |  |  |  |  |  |  |  |  |  |  |  |  |
| I300 | -0.38 | 0.15 | -0.13 | -0.30 | 0.33 | -0.35 |  |  |  |  |  |  |  |  |  |  |  |  |
| G300 | -0.11 | -0.01 | **0.74** | -0.21 | -0.05 | -0.17 | 0.13 |  |  |  |  |  |  |  |  |  |  |  |
| F300 | 0.46 | 0.17 | -0.20 | 0.48 | -0.03 | 0.40 | **-0.73** | **-0.54** |  |  |  |  |  |  |  |  |  |  |
| A300 | **0.52** | -0.13 | -0.39 | 0.39 | -0.17 | 0.12 | **-0.61** | **-0.53** | **0.69** |  |  |  |  |  |  |  |  |  |
| I500 | -0.35 | 0.18 | -0.18 | -0.30 | 0.32 | -0.28 | **0.94** | 0.13 | **-0.74** | **-0.62** |  |  |  |  |  |  |  |  |
| G500 | -0.28 | -0.15 | **0.57** | -0.32 | -0.17 | -0.37 | 0.23 | **0.85** | **-0.56** | **-0.54** | 0.18 |  |  |  |  |  |  |  |
| F500 | 0.44 | 0.13 | -0.20 | 0.42 | -0.09 | 0.40 | **-0.76** | **-0.53** | **0.99** | **0.70** | **-0.76** | **-0.60** |  |  |  |  |  |  |
| A500 | **0.54** | -0.13 | -0.31 | 0.38 | -0.17 | 0.13 | **-0.64** | -0.49 | **0.68** | **0.97** | **-0.66** | -0.48 | **0.68** |  |  |  |  |  |
| I1000 | **-0.52** | -0.04 | -0.13 | -0.43 | 0.19 | -0.37 | **0.91** | 0.14 | **-0.79** | **-0.70** | **0.93** | 0.29 | **-0.83** | **-0.71** |  |  |  |  |
| G1000 | -0.20 | -0.07 | 0.37 | -0.12 | 0.04 | -0.28 | 0.21 | **0.58** | **-0.53** | -0.36 | 0.17 | **0.81** | **-0.59** | -0.30 | 0.27 |  |  |  |
| F1000 | 0.43 | 0.14 | -0.12 | 0.40 | -0.09 | 0.38 | **-0.74** | -0.46 | **0.96** | **0.63** | **-0.79** | **-0.53** | **0.98** | **0.62** | **-0.84** | **-0.58** |  |  |
| A1000 | 0.43 | -0.24 | -0.33 | 0.23 | -0.34 | 0.09 | **-0.68** | -0.46 | **0.61** | **0.92** | **-0.67** | -0.40 | **0.61** | **0.96** | **-0.69** | -0.21 | **0.54** |  |
| GB300 | **-0.62** | -0.32 | 0.15 | **-0.66** | -0.18 | -0.28 | **0.54** | 0.31 | **-0.72** | **-0.74** | **0.60** | 0.32 | **-0.71** | **-0.68** | **0.75** | 0.14 | **-0.70** | **-0.58** |
| FB300 | 0.38 | 0.17 | -0.06 | 0.41 | -0.07 | 0.40 | **-0.70** | -0.42 | **0.95** | **0.54** | **-0.76** | -0.49 | **0.96** | **0.53** | **-0.80** | **-0.58** | **0.99** | 0.45 |
| WB300 | -0.35 | -0.30 | 0.08 | -0.19 | 0.14 | -0.22 | 0.38 | 0.19 | -0.47 | -0.44 | 0.36 | 0.04 | -0.46 | -0.45 | **0.50** | -0.04 | -0.45 | -0.49 |
| AB300 | **0.58** | 0.07 | -0.18 | **0.58** | 0.02 | 0.14 | **-0.56** | -0.41 | **0.74** | **0.83** | **-0.61** | -0.34 | **0.69** | **0.85** | **-0.68** | -0.28 | **0.67** | **0.77** |
| PB300 | 0.37 | 0.12 | 0.29 | 0.30 | -0.05 | 0.12 | -0.38 | 0.01 | 0.47 | 0.30 | **-0.59** | 0.05 | 0.46 | 0.36 | **-0.57** | -0.16 | **0.58** | 0.29 |
| GB500 | **-0.85** | -0.29 | 0.05 | **-0.64** | -0.17 | **-0.58** | **0.60** | 0.32 | **-0.76** | **-0.75** | **0.58** | 0.46 | **-0.77** | **-0.72** | **0.77** | 0.41 | **-0.73** | **-0.65** |
| FB500 | 0.46 | 0.19 | -0.19 | 0.49 | -0.02 | 0.38 | **-0.71** | **-0.52** | **0.99** | **0.69** | **-0.74** | **-0.54** | **0.98** | **0.66** | **-0.80** | **-0.54** | **0.97** | **0.58** |
| WB500 | -0.32 | -0.22 | 0.16 | -0.12 | 0.14 | -0.19 | 0.30 | 0.28 | -0.46 | -0.45 | 0.29 | 0.06 | -0.44 | -0.47 | 0.39 | -0.05 | -0.41 | **-0.51** |
| AB500 | **0.60** | -0.07 | -0.31 | **0.51** | -0.06 | 0.21 | **-0.66** | -0.48 | **0.74** | **0.92** | **-0.62** | -0.46 | **0.72** | **0.94** | **-0.69** | -0.30 | **0.65** | **0.90** |
| PB500 | 0.45 | 0.05 | 0.28 | 0.40 | -0.04 | 0.21 | -0.43 | -0.06 | **0.50** | 0.42 | **-0.61** | 0.03 | 0.47 | 0.47 | **-0.56** | -0.13 | **0.54** | 0.41 |
| GB1000 | **-0.55** | -0.18 | 0.20 | -0.33 | 0.08 | -0.44 | **0.52** | 0.44 | **-0.83** | **-0.64** | 0.49 | **0.55** | **-0.85** | **-0.59** | **0.64** | **0.71** | **-0.81** | **-0.52** |
| FB1000 | 0.42 | 0.22 | -0.07 | 0.40 | -0.05 | 0.32 | **-0.66** | -0.39 | **0.93** | **0.56** | **-0.77** | -0.41 | **0.93** | **0.57** | **-0.80** | -0.49 | **0.97** | 0.48 |
| WB1000 | -0.09 | 0.25 | 0.30 | -0.02 | 0.25 | -0.00 | 0.37 | **0.52** | -0.45 | **-0.55** | 0.46 | 0.20 | -0.44 | **-0.62** | 0.36 | -0.10 | -0.41 | **-0.65** |
| AB1000 | 0.47 | -0.11 | -0.36 | 0.38 | -0.18 | 0.22 | **-0.75** | **-0.56** | **0.77** | **0.89** | **-0.70** | **-0.57** | **0.78** | **0.91** | **-0.74** | -0.41 | **0.73** | **0.89** |
| PB1000 | 0.46 | 0.09 | 0.19 | 0.41 | -0.03 | 0.31 | -0.49 | -0.19 | **0.61** | 0.48 | **-0.60** | -0.07 | **0.55** | **0.53** | **-0.54** | -0.11 | **0.55** | 0.48 |
| Age | -0.30 | -0.04 | 0.21 | -0.41 | 0.22 | -0.09 | **0.81** | 0.36 | **-0.77** | **-0.77** | **0.79** | 0.45 | **-0.76** | **-0.76** | **0.83** | 0.34 | **-0.73** | **-0.75** |
| Size | 0.02 | 0.06 | 0.36 | -0.12 | -0.22 | 0.11 | -0.44 | 0.38 | 0.02 | 0.03 | -0.37 | 0.29 | 0.06 | 0.04 | -0.38 | 0.47 | 0.03 | 0.14 |

Continued…

|  | GB300 | FB300 | WB300 | AB300 | PB300 | GB500 | FB500 | WB500 | AB500 | PB500 | GB1000 | FB1000 | WB1000 | AB1000 | PB1000 | Age |
| --- | --- | --- | --- | --- | --- | --- | --- | --- | --- | --- | --- | --- | --- | --- | --- | --- |
| FB300 | **-0.66** |  |  |  |  |  |  |  |  |  |  |  |  |  |  |  |
| WB300 | **0.63** | -0.43 |  |  |  |  |  |  |  |  |  |  |  |  |  |  |
| AB300 | **-0.74** | **0.63** | -0.46 |  |  |  |  |  |  |  |  |  |  |  |  |  |
| PB300 | -0.46 | **0.58** | -0.32 | **0.63** |  |  |  |  |  |  |  |  |  |  |  |  |
| GB500 | **0.82** | **-0.69** | **0.59** | **-0.73** | -0.47 |  |  |  |  |  |  |  |  |  |  |  |
| FB500 | **-0.75** | **0.96** | -0.47 | **0.76** | **0.53** | **-0.77** |  |  |  |  |  |  |  |  |  |  |
| WB500 | **0.55** | -0.39 | **0.97** | -0.45 | -0.26 | **0.53** | -0.45 |  |  |  |  |  |  |  |  |  |
| AB500 | **-0.70** | **0.58** | -0.48 | **0.88** | 0.29 | **-0.74** | **0.72** | **-0.50** |  |  |  |  |  |  |  |  |
| PB500 | **-0.51** | **0.54** | -0.35 | **0.71** | **0.93** | **-0.54** | **0.55** | -0.32 | 0.40 |  |  |  |  |  |  |  |
| GB1000 | **0.58** | **-0.80** | 0.49 | **-0.64** | -0.44 | **0.85** | **-0.84** | 0.47 | **-0.62** | -0.47 |  |  |  |  |  |  |
| FB1000 | **-0.71** | **0.96** | -0.45 | **0.69** | **0.71** | **-0.71** | **0.95** | -0.41 | **0.57** | **0.66** | **-0.78** |  |  |  |  |  |
| WB1000 | 0.41 | -0.36 | **0.58** | -0.45 | -0.19 | 0.25 | -0.41 | **0.69** | **-0.58** | -0.25 | 0.19 | -0.38 |  |  |  |  |
| AB1000 | **-0.64** | **0.65** | -0.46 | **0.77** | 0.24 | **-0.67** | **0.74** | -0.48 | **0.95** | 0.32 | **-0.61** | **0.62** | **-0.63** |  |  |  |
| PB1000 | **-0.52** | **0.54** | -0.39 | **0.69** | **0.75** | **-0.60** | **0.63** | -0.39 | 0.45 | **0.91** | **-0.55** | **0.66** | -0.32 | 0.37 |  |  |
| Age | **0.67** | **-0.68** | 0.29 | **-0.74** | -0.40 | **0.61** | **-0.76** | 0.20 | **-0.74** | -0.46 | **0.54** | **-0.69** | 0.30 | **-0.78** | **-0.51** |  |
| Size | -0.21 | -0.02 | -0.33 | -0.16 | -0.06 | -0.13 | -0.00 | -0.24 | -0.04 | -0.09 | 0.11 | 0.03 | -0.14 | 0.01 | 0.03 | -0.15 |
